# Dietary fructose-induced gut dysbiosis promotes mouse hippocampal neuroinflammation: a benefit of short-chain fatty acids

**DOI:** 10.1186/s40168-019-0713-7

**Published:** 2019-06-29

**Authors:** Jian-Mei Li, Rong Yu, Li-Ping Zhang, Shi-Yu Wen, Shui-Juan Wang, Xiao-Yang Zhang, Qiang Xu, Ling-Dong Kong

**Affiliations:** State Key Laboratory of Pharmaceutical Biotechnology, School of Life Sciences, Nanjing University, Nanjing, 210023 People’s Republic of China

**Keywords:** Gut dysbiosis, Neuroinflammation, Neuronal loss, Short-chain fatty acids, NLRP6 inflammasome

## Abstract

**Background:**

Western-style diets arouse neuroinflammation and impair emotional and cognitive behavior in humans and animals. Our previous study showed that a high-fructose diet caused the hippocampal neuroinflammatory response and neuronal loss in animals, but the underlying mechanisms remained elusive. Here, alterations in the gut microbiota and intestinal epithelial barrier were investigated as the causes of hippocampal neuroinflammation induced by high-fructose diet.

**Results:**

A high-fructose diet caused the hippocampal neuroinflammatory response, reactive gliosis, and neuronal loss in C57BL/6N mice. Depletion of the gut microbiota using broad-spectrum antibiotics suppressed the hippocampal neuroinflammatory response in fructose-fed mice, but these animals still exhibited neuronal loss. Gut microbiota compositional alteration, short-chain fatty acids (SCFAs) reduction, intestinal epithelial barrier impairment, NOD-like receptor family pyrin domain-containing 6 (NLRP6) inflammasome dysfunction, high levels of serum endotoxin, and FITC-dextran were observed in fructose-fed mice. Of note, SCFAs, as well as pioglitazone (a selective peroxisome proliferator-activated receptor gamma (PPAR-γ) agonist), shaped the gut microbiota and ameliorated intestinal epithelial barrier impairment and NLRP6 inflammasome dysfunction in fructose-fed mice. Moreover, SCFAs-mediated NLRP6 inflammasome activation was inhibited by histamine (a bacterial metabolite) in ex vivo colonic explants and suppressed in *murine CT26 colon carcinoma cells* transfected with *NLRP6* siRNA*.* However, pioglitazone and GW9662 (a PPAR-γ antagonist) exerted no impact on SCFAs-mediated NLRP6 inflammasome activation in ex vivo colonic explants, suggesting that SCFAs may stimulate NLRP6 inflammasome independently of PPAR-γ activation. SCFAs and pioglitazone prevented fructose-induced hippocampal neuroinflammatory response and neuronal loss in mice. Additionally, SCFAs activated colonic NLRP6 inflammasome and increased DCX^+^ newborn neurons in the hippocampal DG of control mice.

**Conclusions:**

Our findings reveal that gut dysbiosis is a critical factor for a high-fructose diet-induced hippocampal neuroinflammation in C57BL/6N mice possibly mediated by impairing intestinal epithelial barrier. Mechanistically, the defective colonic NLRP6 inflammasome is responsible for intestinal epithelial barrier impairment. SCFAs can stimulate NLRP6 inflammasome and ameliorate the impairment of intestinal epithelial barrier, resulting in the protection against a high-fructose diet-induced hippocampal neuroinflammation and neuronal loss. This study addresses a gap in the understanding of neuronal injury associated with Western-style diets. A new intervention strategy for reducing the risk of neurodegenerative diseases through SCFAs supplementation or dietary fiber consumption is emphasized.

**Electronic supplementary material:**

The online version of this article (10.1186/s40168-019-0713-7) contains supplementary material, which is available to authorized users.

## Background

Neuroinflammation is a hallmark of neurodegenerative diseases associated with Western-style diets, including high-calorie diets rich in processed sugars (fructose or sucrose) and fats [1–4]. The gut microbiota has been reported to modulate adult hippocampal neurogenesis and neurological function by controlling the maturation and function of microglia in germ-free and antibiotic-treated specific pathogen-free (SPF) mice [5, 6], indicating that the alteration in the gut microbiota (gut dysbiosis) may be major event initiating neuroinflammation and subsequent neuronal injury [5, 7, 8].

The intestinal epithelial barrier prevents the entry of exterior antigens from the gut lumen into the host, which may exacerbate both local and systemic immune responses [9, 10]. The front line of this barrier is composed of epithelial cells and apical junctional complexes encompassing tight junction (TJ) proteins and adherens junctions between adjacent epithelial cells [11, 12]. NOD-like receptor family pyrin domain-containing 6 (NLRP6) inflammasome, an intracellular innate immune sensor, orchestrates colonic mucosal defense against bacterial pathogens [13–15]. Commensal microbes and their metabolites are crucial for the integrity of the intestinal epithelial barrier [16–18], possibly regulating the NLRP6 inflammasome signaling to maintain intestinal microenvironment homeostasis [19]. Of note, some dietary ingredients, such as fructose, fat, and heme, have been reported to alter the gut microbiota and disrupt intestinal epithelial barrier integrity in mice [20–23]. In particular, diets rich in fructose cause gut dysbiosis, leading to microbial metabolite disorder and intestinal epithelial barrier damage in rodents [20, 23, 24]. Dietary fructose also disrupts hippocampal energy homeostasis, induces neuroinflammation and neuronal injury, and impairs spatial learning and memory in rodents [25–27]. However, it is unclear whether dietary fructose-induced gut dysbiosis promotes hippocampal neuroinflammation through the impairment of intestinal epithelial barrier with the NLRP6 inflammasome dysfunction.

A diet rich in fiber ameliorates intestinal epithelial barrier defect and inflammation in dextran sodium sulfate (DSS)-induced colitic mice [28] and protects mouse lung from allergic inflammation [29]. Ingested dietary fibers are readily fermented by colonic bacteria to produce organic acids, including short-chain fatty acids (SCFAs). Treatment with SCFAs attenuates a high-fat diet-induced change in the gut microbiota and intestinal epithelial barrier integrity in C57BL/6 mice, possibly through the inhibition of systemic inflammation [30, 31]. SCFAs may have the ability to modulate the maturation and function of microglia in the brain, suggesting the potential benefits of gut microbiota-derived SCFAs in protecting against neuroinflammatory process [6, 32]. Neuroprotective effects of SCFAs have also been demonstrated in animal models of neurodegenerative disorders [32, 33], but there is a need for more evidence.

In this study, we found that dietary fructose-driven gut dysbiosis caused intestinal epithelial barrier impairment associated with the occurrence of hippocampal neuroinflammation in C57BL/6N mice. Peroxisome proliferator-activated receptor gamma (PPAR-γ) is a butyrate sensor in the colonic lumen [34]. Microbiota-activated PPAR-γ signaling has been reported to prevent dysbiotic expansion of pathogenic bacteria by driving the energy metabolism of colonic epithelial cells [35]. We also highlighted the key molecular mechanisms by which SCFAs, as well as pioglitazone (a PPAR-γ agonist), repaired intestinal epithelial barrier damage by activating the NLRP6 inflammasome, possibly contributing to the inhibition of hippocampal neuroinflammatory response and neuronal loss in fructose-fed mice.

## Results

### Depletion of the gut microbiota inhibits hippocampal neuroinflammation in fructose-fed mice

**Fig. 1:**
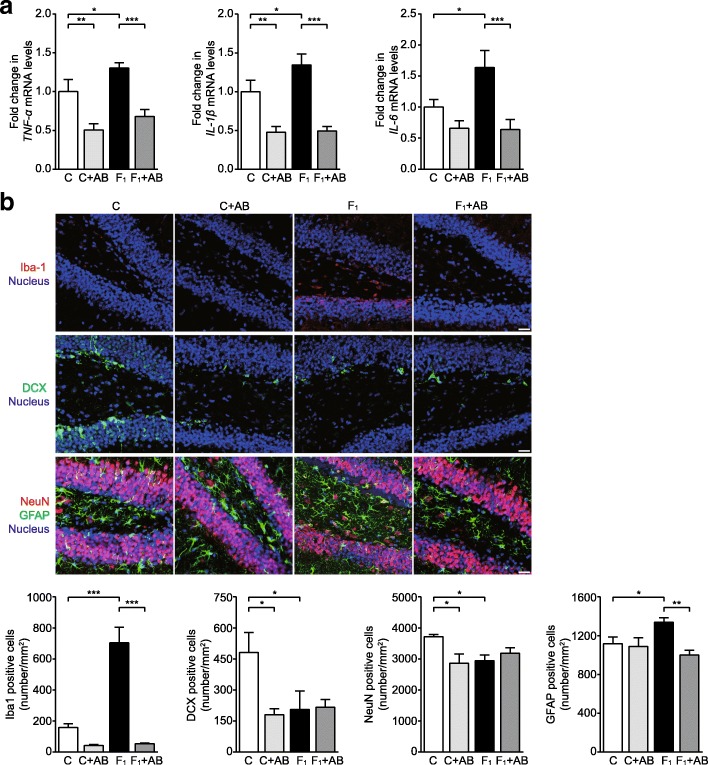
Depletion of the gut microbiota inhibits hippocampal neuroinflammation but fails to reverse the neuronal loss in high-fructose diet-fed C57BL/6N mice. **a** TNF-α, IL-1β, and IL-6 mRNA levels in the hippocampus (*n* = 6). **b** Representative immunofluorescence images and quantitative analysis of Iba-1-positive cells (red), DCX-positive cells (green), NeuN-positive cells (red), and GFAP-positive cells (green) with nuclear counterstain (blue) in the hippocampal DG. Bars, 25 μm. Data are presented as mean ± SEM. *p < 0.05, **p < 0.01, ***p < 0.001 indicate significant difference. C control group, F_1_ eight-week fructose-fed group, AB antibiotics-treated group

A hippocampal neuroinflammatory response, characterized by significant upregulation of interleukin-1 beta (IL-1β), tumor necrosis factor alpha (TNF-α), and IL-6 mRNA levels, was observed in C57BL/6N mice fed a high-fructose diet for eight weeks (Fig. 1a). Immunofluorescence staining showed that the numbers of Iba-1^+^ microglia were significantly increased in the whole hippocampus of high-fructose diet-fed mice compared with standard diet-fed animals (Fig. 1b and Additional file 1: Figure S1a). The numbers of NeuN^+^ neurons and doublecortin (DCX)^+^ newborn neurons were significantly reduced, whereas of GFAP^+^ astrocytes were increased, in the hippocampal dentate gyrus (DG) (Fig. 1b) but not in the cornu ammonis 1 (CA1) and CA3 areas (Additional file 1: Figure S1b) in fructose-fed mice. These results confirm that a high-fructose diet induces hippocampal neuroinflammatory response and neuronal loss in C57BL/6N mice.

To determine whether dietary fructose-induced hippocampal neuroinflammatory response required continuous input from the resident microbes, half of the control and fructose-fed animals were orally treated with broad-spectrum antibiotics (including ampicillin, vancomycin, neomycin, metronidazole, and amphotericin B) during the last four weeks of the experiment. Antibiotic treatment severely reduced microbial abundance and diversity, resulting in a blank microbial composition profile in both the control and fructose-fed groups (Additional file 1: Figure S1c and d). In control mice, antibiotics caused significant reduction in IL-1β and TNF-α mRNA levels and a decreasing trend in IL-6 mRNA levels (Fig. 1a) and the numbers of Iba-1^+^ microglia in the whole hippocampus (Fig. 1b and Additional file 1: Figure S1a). Notably, in fructose-fed mice, antibiotics inhibited the upregulation of hippocampal IL-1β, TNF-α, and IL-6 mRNA levels and the increase in the numbers of Iba-1^+^ microglia (Fig. 1a, b and Additional file 1: Figure S1a). Moreover, antibiotics significantly decreased the numbers of NeuN^+^ neurons and DCX^+^ newborn neurons without affecting the numbers of GFAP^+^ astrocytes in the hippocampal DG in control mice (Fig. 1b). Of note, antibiotics suppressed the increase in the numbers of GFAP^+^ astrocytes but failed to attenuate the fructose-induced decrease in the numbers of NeuN^+^ neurons and DCX^+^ newborn neurons (Fig. 1b) in mice. There were no significant differences in the numbers of GFAP^+^ astrocytes in the hippocampal CA1 and CA3 regions among the groups (Additional file 1: Figure S1b). These results indicate that gut dysbiosis plays an important role in a high-fructose diet-induced hippocampal neuroinflammatory response in C57BL/6N mice.

### A high-fructose diet induces gut dysbiosis, SCFAs reduction, and intestinal epithelial barrier impairment in C57BL/6N mice

**Fig. 2:**
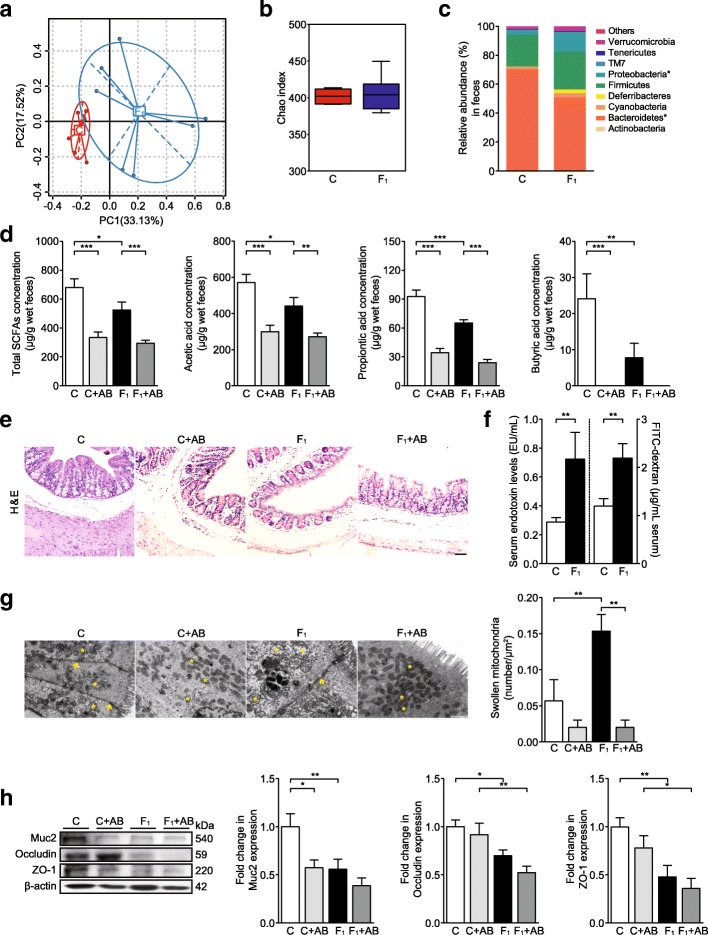
A high-fructose diet induces gut dysbiosis, SCFAs reduction, and colonic epithelial barrier impairment in C57BL/6N mice. **a** PCoA based on the relative abundance of bacterial OTU, **b** Chao1 diversity indexes of bacterial community, **c** relative abundance of bacterial phyla, and **d** SCFA concentrations in fecal samples (*n* = 7). **e** Colon histopathology (bars, 50 μm). **f** Endotoxin and FITC-dextran levels in serum (*n* = 8). **g** Representative transmission electron micrographs of colon epithelial cells (bars, 1 μm). Arrows indicate gap junctions between two neighbored cells. Asterisks indicate the mitochondria in epithelial cells. **h** Immunoblot analysis for protein levels of Muc2, occludin, and ZO-1 in colon tissue (*n* = 6). Quantification: band intensity normalized to β-actin. Data are presented as mean ± SEM. *p < 0.05, **p < 0.01, ***p < 0.001 indicate significant difference. C control group, F_1_ eight-week fructose-fed group, AB antibiotics-treated group

To assess the influence of dietary fructose on the gut microbiota in mice, the fecal microbiota was analyzed using 16S rRNA gene amplicon sequencing. A high-fructose diet feeding for eight weeks changed the microbial community structure but had no effect on microbial alpha-diversity in mice (Fig. 2a and b, Additional file 1: Figure S2a). The abundance of *Bacteroidetes* was significantly decreased and *Proteobacteria* was significantly increased in fructose-fed mice. Moreover, these animals showed an increasing trend in *Firmicutes* (Fig. 2c). Fructose feeding also led to marked enrichment of the pathogenic bacterial taxa *Deferribacteraceae* (*Mucispirillum*) and *Helicobacteraceae* (*Helicobacter*) in mice (Additional file 1: Figure S2b). Although main SCFAs-producing bacteria, such as *Lachnospiraceae* and *Ruminococcaceae*, were highly enriched in fructose-fed mice (Additional file 1: Figure S2c), the fecal concentrations of acetate, propionate, butyrate, and total SCFAs were significantly lower in fructose-fed mice than in control group (Fig. 2d). Antibiotics reduced the fecal concentrations of acetate, propionate, butyrate, and total SCFAs in both control and fructose-fed animals (Fig. 2d).

A high-fructose diet feeding caused histological changes characterized by thinning of the intestinal mucosa, epithelium, and muscularis mucosae; loss of crypts and glands, edema of the lamina propria; and discrete infiltration of inflammatory cells in mice (Fig. 2e and Additional file 1: Figure S2d). Consistently, serum endotoxin and FITC-dextran levels were increased significantly in fructose-fed mice compared with control animals (Fig. 2f). Electron microscopy also showed short and sparse microvilli, severe mitochondrial swelling, and vacuole change in epithelial cells as well as loss of TJ domains, irregular gaps, and increased distance between adjacent epithelial cells in the colon and distal ileum in fructose-fed mice (Fig. 2g and Additional file 1: Figure S2f). Moreover, colonic mucin 2 (Muc2), occludin, and zonula occludens-1 (ZO-1) protein levels were downregulated significantly in fructose-fed mice (Fig. 2h). After antibiotic treatment, both control and fructose-fed mice showed intestinal histological changes, but intestinal epithelial cells were arranged tightly and in an orderly manner with perfect mitochondrial structure (Fig. 2e, g, Additional file 1: Figure S2d and f). In addition, fructose-fed mice showed a significant decrease in serum endotoxin levels after antibiotic treatment (Additional file 1: Figure S2e), although there was still significantly decreased colonic Muc2, occludin, and ZO-1 expression in the fructose-fed group compared with the control group (Fig. 2h). In control mice, antibiotics caused a significant decrease in colonic Muc2 expression and a trend toward a decrease in colonic occludin and ZO-1 expression (Fig. 2h). Together, these results indicate that a high-fructose diet induces gut dysbiosis, reduces SCFAs levels, and causes intestinal epithelial barrier impairment in C57BL/6N mice.

### SCFAs and pioglitazone partially shape gut dysbiosis and ameliorate intestinal epithelial barrier impairment in fructose-fed mice

**Fig. 3:**
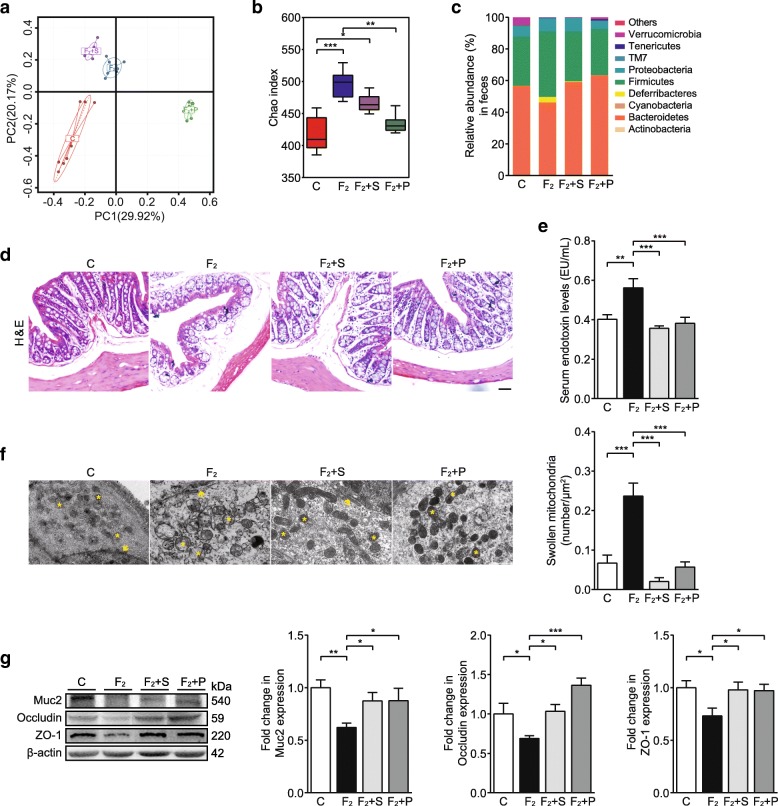
SCFAs and pioglitazone shape gut dysbiosis and improve intestinal epithelial barrier impairment in high-fructose diet-fed C57BL/6N mice. **a** PCoA based on the relative abundance of bacterial OTU, **b** Chao1 diversity indexes of bacterial community, and **c** relative abundance of bacterial phyla in fecal samples (*n* = 7). **d** Colon histopathology (bars, 50 μm). **e** Endotoxin levels in serum (*n* = 8). **f** Representative transmission electron micrographs of colonic epithelial cells (bars, 1 μm). Arrows indicate gap junctions between two neighbored cells. Asterisks indicate the mitochondria in epithelial cells. **g** Immunoblot analysis for protein levels of Muc2, occludin, and ZO-1 in colon tissue (*n* = 6). Quantification: band intensity normalized to β-actin. Data are presented as mean ± SEM. *p < 0.05, **p < 0.01, ***p < 0.001 indicate significant difference. C control group, F_2_ 12-week fructose-fed group, S SCFAs-treated group, P pioglitazone-treated group

Next, we tested whether oral administration of SCFAs and a PPAR-γ agonist prevented dietary fructose-induced intestinal epithelial barrier impairment in C57BL/6N mice. Although the microbial structures in fructose-fed mice were still different from those in control mice, to some extent, SCFAs and pioglitazone reduced the enriched diversity in fructose-fed mice (Fig. 3a, b). SCFAs and pioglitazone improved bacterial composition and structure (Fig. 3c and Additional file 1: Figure S3a) and prevented the increase in the abundance of the pathogenic bacterial taxon *Deferribacteraceae* (*Mucispirillum*), but the former failed to decrease the abundance of *Helicobacteraceae* (*Helicobacter*) (Additional file 1: Figure S3b) in fructose-fed mice. In addition, SCFAs and pioglitazone alleviated the pathological changes in the intestinal mucosa, epithelium, and muscularis mucosae and prevented substructural damage to epithelial cells in fructose-fed mice (Fig. 3d, f, Additional file 1: Figure S3c and d). Consistently, the increase in blood endotoxin levels and the reduction in Muc2, ZO-1, and occludin protein expression in fructose-fed mice were also reversed by SCFAs and pioglitazone (Fig. 3e, g). SCFAs-treated normal mice showed no significant change in intestinal structure or colonic occludin expression (Additional file 1: Figure S3e and f). These results support the notion that SCFAs and PPAR-γ agonist pioglitazone shaped gut dysbiosis and prevented intestinal epithelial barrier impairment in fructose-fed mice.

### Fructose-fed mice exhibit a defect in colonic NLRP6 inflammasome, which is ameliorated by SCFAs and pioglitazone

**Fig. 4:**
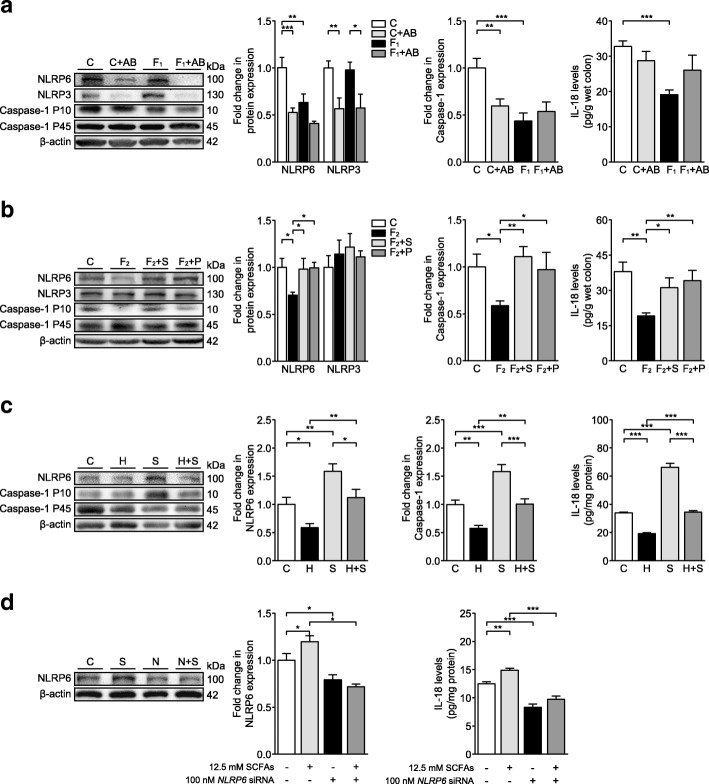
A high-fructose diet causes colonic NLRP6 inflammasome dysfunction in C57BL/6N mice, which is ameliorated by SCFAs. Immunoblot analysis of NLRP6, NLRP3, and caspase-1 P10/P45 and IL-18 production (**a**, **b**) in colonic tissues of mice (*n* = 6), **c** cultured ex vivo colonic explants (*n* = 6), and **d** cultured mouse colon CT26 cells from three independent experiments. Data are presented as mean ± SEM. *p < 0.05, **p < 0.01, ***p < 0.001 indicate significant difference. C control group, F_1_ eight-week fructose-fed group, F_2_ 12-week fructose-fed group, AB antibiotics-treated group, S SCFAs-treated group, P pioglitazone-treated group, H histamine-treated group, G GW9662-treated group

Notably, the abnormal production of colonic interferon gamma (IFN-γ), TNF-α, and IL-10 further demonstrated a gut immune imbalance in fructose-fed mice, which were ameliorated by SCFAs and pioglitazone (Additional file 1: Figure S4a). Colonic NLRP6 and cleaved caspase-1 P10 levels were significantly reduced, and colonic IL-18 secretion was decreased in fructose-fed mice (Fig. 4a). In the control group, antibiotics significantly decreased NLRP6 and cleaved caspase-1 P10 protein levels but showed no effect on colonic IL-18 secretion (Fig. 4a). In fructose-fed mice, antibiotics reversed the decrease in colonic IL-18 secretion and further suppressed colonic NLRP6 expression (Fig. 4a). SCFAs or pioglitazone also prevented the reduction in colonic NLRP6 levels and caspase-1 activation in fructose-fed mice, resulting in the recovery of IL-18 production (Fig. 4b). In comparison to that in control mice, colonic NLRP3 expression was not altered in fructose-fed mice treated with SCFAs and pioglitazone but was decreased significantly in antibiotic-treated control and fructose-fed mice (Fig. 4a, b). Moreover, colonic expression of PPAR-γ and its target gene (*ANGPTL4*) was downregulated in fructose-fed mice, which were restored by SCFAs and pioglitazone (Additional file 1: Figure S4b and c).

In the colons of normal mice, oral administration of SCFAs upregulated NLRP6 expression and increased IL-18 production (Additional file 1: Figure S4d). In an ex vivo system of cultured mouse colonic explants (ex vivo colonic explants), SCFAs also increased NLRP6 expression and cleaved caspase-1 P10 protein levels and promoted IL-18 production, and these effects were suppressed by histamine (a bacterial metabolite) (Fig. 4c). Additionally, NLRP6 protein levels and IL-18 production were increased by SCFAs but decreased by *NLRP6* siRNAs in murine CT26 colon carcinoma cells (Fig. 4d). However, neither pioglitazone nor GW9662 (a PPAR-γ antagonist) affected basal or SCFAs-stimulated NLRP6 inflammasome activation in the ex vivo colonic explants (Additional file 1: Figure S4e). These results suggest that SCFAs may activate the NLRP6 inflammasome independently of PPAR-γ activation.

### SCFAs and pioglitazone suppress the hippocampal neuroinflammatory response and neuronal loss in fructose-fed mice

**Fig. 5:**
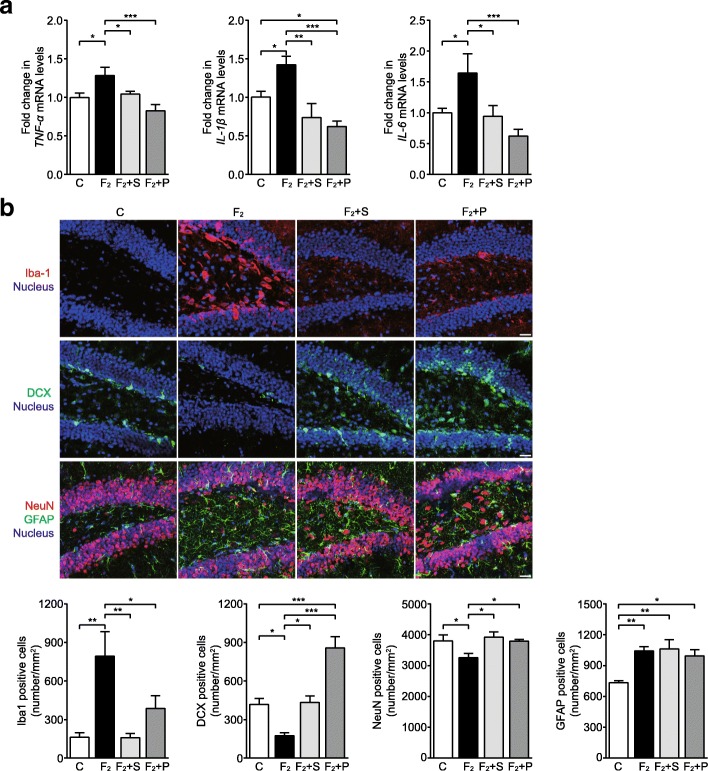
SCFAs and pioglitazone inhibit hippocampal neuroinflammation and protect against neuronal loss in high-fructose diet-fed C57BL/6N mice. **a** TNF-α, IL-1β, and IL-6 mRNA levels in the hippocampus (*n* = 6). **b** Representative immunofluorescence images and quantitative analysis of Iba-1-positive cells (red), DCX-positive cells (green), NeuN-positive cells (red), and GFAP-positive cells (green) with nuclear counterstain (blue) in the hippocampal DG. Bars, 25 μm. Data are presented as mean ± SEM. *p < 0.05, **p < 0.01, ***p < 0.001 indicate significant difference. C control group, F_2_ 12-week fructose-fed group, S SCFAs-treated group, P pioglitazone-treated group

We confirmed that oral treatment with SCFAs significantly reduced IL-1β, TNF-α, and IL-6 mRNA levels and the numbers of Iba-1^+^ microglia in the hippocampus of fructose-fed mice (Fig. 5a, b and Additional file 1: Figure S5a). SCFAs clearly increased the numbers of DCX^+^ newborn neurons and NeuN^+^ neurons (Fig. 5b) but could not inhibit the increase in the numbers of GFAP^+^ astrocytes in the DG of the hippocampus in fructose-fed mice (Fig. 5b). More interestingly, SCFAs significantly increased the numbers of DCX^+^ newborn neurons in the DG of the hippocampus in control mice (Additional file 1: Figure S5c). Similarly, pioglitazone significantly decreased IL-1β, TNF-α, and IL-6 mRNA levels and the numbers of Iba-1^+^ microglia in the hippocampus of fructose-fed mice (Fig. 5a, b and Additional file 1: Figure S5a). Moreover, pioglitazone clearly increased the numbers of DCX^+^ newborn neurons and NeuN^+^ neurons in hippocampal DG in fructose-fed mice (Fig. 5b). The numbers of newborn neurons in fructose-fed mice treated with pioglitazone were even greater than that in control mice (Fig. 5b). Pioglitazone also failed to change the increase in the numbers of GFAP^+^ astrocytes in the hippocampus of fructose-fed mice (Fig. 5b). There were no significant differences in the numbers of GFAP^+^ astrocytes in the hippocampal CA1 and CA3 regions among fructose-fed mice treated or not treated with SCFAs and pioglitazone (Additional file 1: Figure S5b). These results suggest that SCFAs and pioglitazone can prevent neuronal injury by inhibiting neuroinflammation in fructose-fed mice.

## Discussion

In the current study, we provided new experimental evidence for a critical role of a high-fructose diet-driven gut dysbiosis in the impairment of intestinal epithelial barrier that triggers hippocampal neuroinflammation and neuronal loss in mice. We found that C57BL/6N mice fed a high-fructose diet for 8 or 12 weeks exhibited clear hippocampal neuroinflammatory response, and the activation and gliosis of microglia and astrocytes simultaneously caused significant reduction in the numbers of total and newborn neurons in hippocampal DG. The hippocampal DG is one of the few brain structures with high rates of neurogenesis. This brain structure is vulnerable to neuroinflammation, which causes neuronal loss and functional impairment [36]. Our findings demonstrated that a high-fructose diet induced early signs of neurodegeneration in hippocampus, representing a risk factor for the onset of neurodegenerative diseases [4]. Unexpectedly, fructose-fed C57BL/6N mice did not exhibit clear memory impairment in preliminary new objective recognition (NOR) and Morris water maze (MWM) tests, nor did they exhibit any changes in weight gain and insulin resistance compared with control mice (Additional file 1: Figure S6a–f). Previously, C57BL/6 mice fed with 15% fructose solution for eight weeks are reported to show spatial memory impairment in the MWM test [37]. Inconsistent with our findings, rats fed with 10% fructose solution for 28 weeks in a previous study show significantly reduced discrimination indices in the NOR test, whereas no spatial memory loss is observed in the MWM test in the fructose-fed group compared with the control group [38]. In another report, rats fed with a high-fructose diet (55% kcal/g from fructose) for 10 weeks also show no spatial memory loss in the MWM test [39]. These conflicting results may be due to the differences in the amounts of fructose ingested and/or to different susceptibilities of the strains of animals used to the harmful effects of high-fructose diets [40].

One of the most striking observations following broad-spectrum antibiotic treatment was the inhibition of hippocampal neuroinflammation and reactive gliosis in a high-fructose diet-fed group, which was similar to the action of antibiotics in a murine model of Alzheimer’s disease, a progressive neurodegenerative disease [41]. We also found that the depletion of the gut microbiota caused a hippocampal neuronal loss in both the control and fructose-fed groups, suggesting the critical role of gut microbes and/or their metabolites in the modulation of hippocampal neurogenesis. Interestingly, SCFAs or the gut microbiota can restore defective microglia and reinforce the integrity of the blood-brain barrier in germ-free mice [6, 42]. The beneficial effects of SCFAs on hippocampal neuroinflammation and neuronal loss in fructose-fed animals further demonstrate the critical role of commensal microbes and/or their metabolites in the modulation of neurological function [7].

A critical objective was to determine whether a high-fructose diet could alter the gut microbial structure and SCFAs production in C57BL/6N mice. The changes at the phylum levels were consistent with a significantly lower relative abundance of *Bacteroidetes* and a significantly higher relative abundance of *Proteobacteria* in the fructose-fed group than in the control group [23]. Similar changes in the gut microbiota have been observed in humans and animals with inflammatory bowel disease [43] and neurodegeneration [44]. Increased *Mucispirillum* abundance is a signature of early disruption of the colonic surface mucus layer [45]. The increased abundance of pathogenic bacteria suggests the existence of infection and inflammation in fructose-fed C57BL/6N mice. Major SCFA-producing bacteria were highly enriched in fructose-fed animals in our study, while these bacteria were previously reduced in C57BL/6 J mice fed an 8% fructose solution for 12 weeks [46]. The contradictory findings may be due to the different genetic background of the animals. Additionally, our data demonstrated that fructose-driven gut dysbiosis contributed to intestinal epithelial barrier damage in C57BL/6N mice. Importantly, antibiotic-induced gut dysbiosis significantly reduced SCFAs production and downregulated colonic TJ expression in both control and fructose-fed groups. SCFAs exert protective effects on intestinal homeostasis and intestinal barrier function by modulating the inflammatory response against infectious bacteria in rodents [47, 48]. SCFAs ameliorated fructose-induced damage to the intestinal epithelial barrier, resulting in the reduction of systemic endotoxin levels in C57BL/6N mice. These results further suggest that gut microbes and their metabolites are the crucial supports for intestinal epithelial barrier integrity, which may help to explain the inhibitory effects of SCFAs on hippocampal neuroinflammation and neuronal loss in high-fructose diet-fed animals.

The NLRP6 inflammasome-derived IL-18 regulates colonic antimicrobial peptide expression [19] and maintains mucus layers [13, 15], orchestrating host defense against bacterial pathogens [49]. NLRP6-deficient C57BL/6 mice exhibit dramatic decreases in the diversity and richness of the gut microbiota but significant increase in *Akkermansia muciniphila* colonization [50]. Water-avoidance stress causes colonic NLRP6 inflammasome inhibition and small-bowel inflammation in C57BL/6 mice [51]. Like the NLRP6 inflammasome, the NLRP3 inflammasome is also involved in the innate immune system that discriminates pathogenic bacteria from commensal bacteria and shapes microbial ecology [52]. Here, the downregulation of colonic NLRP6 and NLRP3 expression was not completely consistent with the unchanged IL-18 production in antibiotic-treated C57BL/6N mice, indicating that additional inflammasomes, such as absent in melanoma 2 (AIM2) and/or NLRP4 [52], may be involved in sensing microbes and modulating the gut immune defense system in these animals. In addition, the activation of the colonic NLRP6 inflammasome, rather than the NLRP3 inflammasome, was disrupted, resulting in the reduction of colonic IL-18 production in fructose-fed mice, which were consistent with the increase in pathogenic bacteria and the damage to the intestinal epithelial barrier. SCFA treatment attenuated the reduced NLRP6 and cleaved caspase-1 P10 expression as well as IL-18 production but exerted no impact on NLRP3 expression in fructose-fed mice. SCFAs-induced NLRP6 inflammasome activation was suppressed by histamine in ex vivo colonic explants, and inhibited by *NLRP6* siRNA in CT26 cells, demonstrating a direct regulation of the NLRP6 inflammasome by SCFAs. These results suggest that in addition to the shaping of the microbial composition, the activation of the NLRP6 inflammasome may be another mechanism by which SCFAs protect against a high-fructose diet-induced intestinal epithelial barrier impairment in C57BL/6N mice.

Mechanistically, our results suggest that SCFAs could activate the normal or fructose-impaired NLRP6 inflammasome independently of PPAR-γ activation. Host colonic epithelial PPAR-γ is a direct target of SCFAs, which induce *Angptl4* mRNA expression and protein secretion in colon cells by activating PPAR-γ [53]. Consistent with the reduction in SCFAs, colonic PPAR-γ protein and *Angptl4* gene were downregulated in fructose-fed mice, which were restored by oral administration of SCFAs. PPAR-γ agonist can prevent colonic NLRP6 inflammasome inhibition and intestinal disorder in water-avoidance stress-stimulated C57BL/6 mice [51]. Pioglitazone could improve defective colonic NLRP6 inflammasomes and protect against intestinal epithelial barrier impairment in fructose-fed mice, but it did not impact the activation of the NLRP6 inflammasome in ex vivo colonic explants. These observations suggest that pioglitazone may indirectly improve defective colonic NLRP6 inflammasome, possibly by modulating a high-fructose diet-induced gut dysbiosis, as we found in C57BL/6N mice. Moreover, pioglitazone was found to suppress the neuroinflammatory response and ameliorate neuronal loss in the hippocampus in fructose-fed animals. Pioglitazone has been reported to prevent Western-style diet-induced inflammatory reaction in adipose tissue as well as in the liver and brain in an animal model of obesity [27, 54, 55]. Therefore, pharmacological agents that are capable of preventing gut dysbiosis and the NLRP6 inflammasome dysfunction may have beneficial effects in attenuating a high-fructose diet-induced intestinal epithelial barrier damage and hippocampal neuroinflammation by potentially targeting PPAR-γ.

## Conclusions

We have clearly shown that a high-fructose diet-induced aberrant structure of the gut microbiota and reduced fecal SCFAs levels, demonstrating a contribution of gut dysbiosis to hippocampal neuroinflammation in C57BL/6N mice. Mechanistically, colonic NLRP6 inflammasome dysfunction was involved in intestinal epithelial barrier impairment, which was improved by SCFAs or pioglitazone. These results provide new evidence for the protective mechanisms of SCFAs and pioglitazone against hippocampal neuroinflammatory response and neuronal loss in this animal model. Our findings highlight the adverse impact of a feature of Western-style diets on hippocampal neuroinflammation and suggest a new intervention strategy for neurological dysfunction. However, more direct evidence should be obtained in the future.

## Materials and methods

### Animals and treatments

Four-week-old male C57BL/6N mice (17-20 g) raised under SPF conditions were purchased from the Beijing Vital River Laboratory Animal Technology Co., Ltd. (Beijing, China; production license: SCXK (Su) 2016-0003) and allowed to acclimatize to the animal facility environment for a week before being used for experimentation. They were housed in a specific pathogen-free, temperature- and humidity-controlled environment (22 ± 2 °C, 50 ± 5% humidity) with a standard 12 h light/dark cycle. These mice were given access to food and water ad libitum.

The mice were fed a standard diet (control group, 30 mice) or a high-fructose diet (fructose-fed group, 30 mice) with normal drinking water for four weeks firstly before any treatment in experiments I and II. The total calories were 3.4 kcal/g for the standard diet (rodent diet, XT002, Jiangsu Synergy Pharmaceutical Biological Engineering Co., Ltd., Nanjing, China) and 3.7 kcal/g for a high-fructose diet (35% fructose-derived calories). The high-fructose diet was made with standard mouse chow by adding approximately 30% fructose according to a modified formula based on Choi’s report [56]. Starting from the 4th week, the control and fructose-fed groups in experiments I and II were treated in a different way. In experiment I, half of the control and fructose-fed mice were treated orally with broad-spectrum antibiotics for the last four weeks according to Gacias’ report [57]. The mice were given ampicillin (1 g/L) in drinking water and a cocktail of vancomycin (50 mg/kg), neomycin (100 mg/kg), metronidazole (100 mg/kg), and amphotericin B (1 mg/kg) by gavage once daily. All antibiotics were obtained from Solarbio Science & Technology Co., Ltd. (Beijing, China). In experiment II, fructose-fed mice were divided into three subgroups (15 mice/group) in the fourth week. Two groups were fed the high-fructose diet and simultaneously treated with SCFAs or pioglitazone (a PPAR-γ agonist) for the last eight weeks according to our preliminary study, respectively. Another group continued to be fed the high-fructose diet for the last eight weeks. An admixture (3:1:1 ratio) of sodium acetate (S2889; Sigma-Aldrich Co., Ltd, Shanghai, China), sodium propionate (P1880; Sigma-Aldrich), and sodium butyrate (303410; Sigma-Aldrich) was incorporated into a high-fructose diet at a proportion of 5% (wt/wt). A dose of 30 mg/kg pioglitazone was administered orally to the mice by gavage once a day. Body weight was recorded once a week throughout the experiment.

### In vivo permeability assay

Intestinal permeability was assessed with an in vivo FITC-dextran (FD4; Sigma-Aldrich) permeability assay, as described previously [21]. Mice fasted for 4 h were gavaged with 0.6 mg/g body weight FITC-dextran (4 kDa) in a 25 mg/mL solution, and blood was collected by submandibular bleeding after 3 h. The fluorescence intensity in serum was measured using a fluorescence spectrophotometer (Synergy 2, Biotek, Winooski, VT). FITC-dextran concentrations were determined from a standard curve generated with serial dilutions of FITC-dextran.

### Blood and tissue sample collection

At the end of the experiments, the mice were fasted overnight and anesthetized with sodium pentobarbital (50 mg/kg, i.p.), and blood was obtained by cardiopuncture. These blood samples were centrifuged at 2500 rpm for 10 min to collect the serum samples, which were immediately frozen at − 80 °C for biochemical assays. Hippocampus and colon tissues were dissected for histopathology, transmission electron microscopy (TEM) analysis, immunofluorescence staining, quantitative polymerase chain reaction (qPCR), and Western blot analysis.

### 16S rRNA gene sequence analyses

Fresh stool samples from mice were collected in disinfected tubes, immediately frozen in liquid nitrogen upon collection, and stored at − 80 °C until analysis. Stool DNA samples were isolated, and the microbiomes were analyzed at the Beijing Genomics Institute on an Illumina MiSeq platform. High-quality reads for bioinformatics analysis were selected, and all of the valid reads from all samples were clustered into operational taxonomic units (OTUs) based on 97% sequence similarity. α-Diversity was calculated based on the Chao1 diversity index. The variation between the experimental groups (β-diversity) was assessed with principal coordinate analysis (PCoA) plots. Linear discriminant analysis coupled with effect size (LEfSe) was performed with LEFSE software.

### Determination of fecal SCFAs concentrations

SCFAs in the frozen fecal samples were determined by gas chromatography. The method used was adapted from that of Frost et al. [58]. Briefly, 150–200 mg of feces was combined with 550 μL of PBS. These samples were vortexed thoroughly and centrifuged at 7200 rpm for 10 min, and the supernatant was collected. The SCFAs were extracted through the addition of 250 μL of concentrated hydrochloric acid and 1 mL of diethyl ether followed by vortex mixing for 1 min. The samples were centrifuged at 7200 rpm for 10 min after 12 h, and the ether layer was transferred to a separate capped vial. Gas chromatography was performed on an HP 6890 Plus gas chromatograph equipped with a flame ionization detector, split/splitless injector, and an HP-INNOWax column (30 m length × 0.25 mm inner diameter, 0.25 μm film thickness; Agilent Technologies, USA). The injector and detector temperatures were 300 °C, and the column temperature was programmed to increase from 80 °C for 1 min to 150 °C at 5 °C per min. Nitrogen served as the carrier gas, and injections (1 μL) were made in the split mode (10:1 split). The peak areas were recorded, and all subsequent data manipulation was completed using ChemStation Software (Agilent Technologies). Reference standards of acetic acid (A801295; Macklin), propionic acid (P816183; Macklin), and butyric acid (B802731; Macklin) were dissolved in diethyl ether to make the mixed stock solution. The concentrations of SCFAs were calculated by the standard curve method. The reported values were normalized according to the wet weight of the original fecal sample used.

### Ex vivo system of cultured mouse colonic explants

An ex vivo system of cultured mouse colonic explants was constructed according to the methods in a previous report [13] (Additional file 2). Colons from eight-week-old normal C57BL/6N mice were longitudinally opened and washed thoroughly by flushing several times with cold HBSS containing 100 U/mL penicillin, 100 μg/mL streptomycin, and 50 μg/mL metronidazole. The colon tissues were cut into approximately 4-mm^2^ fragments, placed on presoaked Gelfoam rafts, and cultured in 1 mL of RPMI 1640 medium containing 0.01% BSA, 200 U/mL penicillin, 200 μg/mL streptomycin, and 1% Fungizone. The colonic explants were incubated in the presence of a mixture (3:1:1 ratio) of sodium acetate, sodium propionate, and sodium butyrate with histamine (H7125; Sigma-Aldrich), pioglitazone, or GW9662 (a PPAR-γ antagonist) for 24 h according to the experimental design. The culture medium and the colonic explants were collected to test the activation of the NLRP6 inflammasome and the transcription of PPAR-γ.

### Cell culture and treatment

*Murine CT26 colon carcinoma cells* were purchased from the Shanghai Institutes for Biological Sciences (Shanghai, China) and grown in 1640 medium supplemented with 10% fetal bovine serum in a humidified atmosphere containing 5% CO_2_ at 37 °C. For the experiments, cells were plated in 6-well plates for 12 h and transfected with *NLRP6* siRNA or negative control siRNA (GenePharma, Shanghai, China) using Lipofectamine 2000 for 6 h. These cells were then incubated in 1640 medium in the presence or absence of a 12.5 mM mixture (3:1:1 ratio) of sodium acetate, sodium propionate, and sodium butyrate for 48 h. The culture medium was collected, and the total cellular protein was extracted for biochemical and Western blot analysis.

### qPCR analysis

Hippocampus and colon tissues were isolated from the experimental mice and homogenized in TRIzol Reagent (Invitrogen), and total RNA was isolated according to the manufacturer’s instructions. RNA (1 μg) was reverse transcribed into complementary DNA using HiScript II Select qRT SuperMix (Vazyme, Nanjing, China). Quantitative polymerase chain reaction was performed using gene-specific primer sets and SYBR Green (Vazyme, Nanjing, China) on a real-time PCR detection system (Bio-Rad). The primer sequences used for amplification were as follows: mTNF-αF (5′-CCC CTT TAT TGT CTA CTC CTC-3′), mTNF-αR (5′-CCC AGC ATC TTG TG TTT C-3′), mIL-1βF (5′-ATT GTG GCT GTG GAG AAG-3′), mIL-1βR (5′-AAG ATG AAG GAA AAG AAG GTG-3′), mIL-6F (5′-GCC TTC CCT ACT TCA CAA-3′), mIL-6R (5′-ACA ACT CTT TTC TCA TTT CCA C-3′), mPPAR-γF (5′-GTC TTG GAT GTC CTC GAT GGG-3′), mPPAR-γR (5′-TTA TGG AGC CTA AGT TTG AGT TTG C-3′), mAngplt4F (5′-ATC TCC GAA GCC ATC CTT GTA-3′), mAngplt4R (5′-CTC TGG GGT CTC CAC CAT TTT-3′), mβ-actinF (5′-CTC TCC CTC ACG CCA TC-3′) and mβ-actinR (5′-ACG CAC GAT TTC CCT CTC-3′). All the primers were provided by GENEray Biotechnology (Shanghai, China). The reaction conditions were 94 °C for 30 s followed by 40 cycles of 95 °C for 5 s and 60 °C for 30 s. Relative expression was calculated using the △△Ct method with *β-actin* serving as the reference housekeeping gene. The expression of each target gene was normalized to *β-actin* expression, and the normalized data were presented as the fold change in gene expression in treated mice compared with control mice.

### Western blot analysis

Colon tissues from experimental mice or cultured ex vivo colonic explants from normal mice were homogenized in ice-cold RIPA buffer containing protease inhibitors. The cleared lysates were obtained by centrifugation at 10,000 rpm at 4 °C for 15 min. Protein quantification was carried out using a bicinchoninic acid (BCA) protein assay kit (Thermo Fisher Scientific, USA) with bovine serum albumin as a standard. Equivalent amounts of protein from each sample were separated by 10% SDS–PAGE and transferred onto polyvinylidene fluoride membranes (Millipore, USA). Subsequently, the membranes were blocked in 5% milk, probed overnight at 4 °C with primary antibodies and then incubated with HRP-conjugated secondary antibodies. The primary antibodies included rabbit anti-Muc2 (ab76774, Abcam), rabbit anti-occludin (ab168986, Abcam), rabbit anti-ZO-1 (H-300, Santa Cruz), rabbit anti-NLRP6 (PA5-21022, Thermo Fisher Scientific), rabbit anti-caspase-1 (sc-514, Santa Cruz), rabbit anti-NLRP3 (#15101, CST), rabbit anti-PPAR-γ (#2435, CST), and mouse anti-β-actin (#4970, CST). The signals were detected with an enhanced chemiluminescence system (Tanon, Shanghai, China). The immunoreactive bands were quantified via densitometry using ImageJ (Version 1.50b, National Institutes of Health, USA) and standardized to β-actin and were expressed as fold changes relative to the control value.

### Ileal and colonic histopathology

Excised ileum and colon tissues were fixed in 4% paraformaldehyde and embedded in paraffin. The blocks were serially cut into 5-μm-thick sections and stained with hematoxylin and eosin (H&E). Histological images were obtained using an Olympus IX53 microscope (Tokyo, Japan), an Olympus DP73 digital camera, and Olympus cellSens imaging software.

### Transmission electron microscopy (TEM) analysis

Excised ileum and colon tissues were extensively washed of fecal matter and fixed in 2.5% glutaraldehyde in PBS. For TEM, the tissue samples were processed with standard protocols at the Analysis Center of Nanjing Medical University (Nanjing, China) and examined using a JEOL JEM-1010 transmission electron microscope (Tokyo, Japan).

### Immunofluorescence staining and morphometric analysis

Three animals from each group were anesthetized and transcardially perfused with ice-cold 4% paraformaldehyde. The brains were excised, fixed in 4% paraformaldehyde overnight, incubated in 20% sucrose/PBS overnight at 4 °C, and incubated in 30% sucrose/PBS overnight at 4 °C. Then, the brain samples were frozen in optimum cutting temperature compound (Sakura Finetek, USA) and coronally cut with a cryostat into 30-μm-thick sections. The frozen sections were blocked with 10% fetal bovine serum in PBS for 1 h at room temperature. The slides were incubated with primary antibodies overnight at 4 °C and then incubated for 30 min at 37 °C with Alexa Fluor-conjugated secondary antibodies (Invitrogen). The primary antibodies included mouse anti-Iba1 (sc-32725, Santa Cruz), mouse anti-NeuN (ab104224; Abcam), rabbit anti-DCX (ab77450; Abcam), and rabbit anti-GFAP (Z0334; Dako). The sections were counterstained with 4,6-diamidino-2-phenylindole (DAPI) for nuclear staining. Images were captured using a Leica TCS SP8 confocal microscope. Six randomly selected brain sections from each animal were used to outline and count immunofluorescence-positive cells in the hippocampal DG, CA1, and CA3 regions with Image-Pro Plus software (Media Cybernetics, USA).

### Statistical analysis

Data are expressed as the mean ± SEM. Statistical analyses were performed with GraphPad Prism Software 6.01 (GraphPad Software, San Diego, USA). Differences were analyzed by one-way analysis of variance (ANOVA) with post hoc tests for multiple group comparisons. *p* values < 0.05 were considered to indicate significance.

## Additional files

Additional file 1: **Figures S1–S6.** Supplemental Figures. (DOCX 729 kb)

Additional file 2: Supplemental Methods. (DOCX 24 kb)

## Data Availability

All 16S rRNA gene sequencing reads data has been deposited to the National Center for Biotechnology Information’s Sequence Read Archive under accession number PRJNA540110.

## Abbreviations

AIM2: Absent in melanoma 2
DCX: Doublecortin
DG: Dentate gyrus
DSS: Dextran sodium sulfate
IFN-γ: Interferon gamma
LEfSe: Linear discriminant analysis coupled with effect size
Muc2: Mucin 2
MWM: Morris water maze
NOR: New objective recognition
OGTT: Oral glucose tolerance test
OTUs: Operational taxonomic units
PcoA: Principal coordinate analysis
PPAR-γ: Peroxisome proliferator-activated receptor gamma
SCFAs: Short-chain fatty acids
SPF: Specific pathogen-free
TEM: Transmission electron microscopy
TJs: Tight junction proteins
TNF-α: Tumor necrosis factor alpha
ZO-1: Zonula occludens-1
NLRP3/4/6: NLR family, pyrin domain-containing 3/4/6
IL-1β/6/10/18: Interleukin-1 beta/6/10/18
CA1/3: Cornu amonis 1/3
DAPI: 4,6-Diamidino-2-phenylindole
