# Supplementary material for: Dietary fructose-induced gut dysbiosis promotes mouse hippocampal neuroinflammation: a benefit of short-chain fatty acids
Source: Microbiome. 2019 Jun 29;7:98. doi: 10.1186/s40168-019-0713-7 (PMC6599330; doi:10.1186/s40168-019-0713-7)
Supplement: Supplementary file 1 — Figures S1–S6. Supplemental Figures. (DOCX 729 kb) [file 40168_2019_713_MOESM1_ESM.docx]

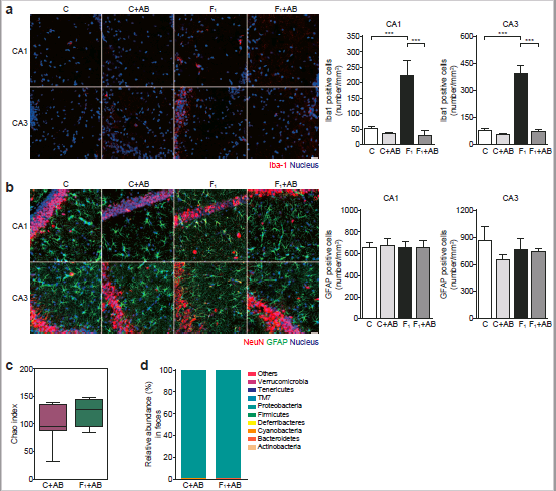


**Additional file 1: Fig S1.** Depletion of the gut microbiota inhibits hippocampal neuroinflammation in a high-fructose diet-fed C57BL/6N mice. Representative immunofluorescence images and quantitative analysis of **(a)** Iba-1 positive cells (red) and **(b)** NeuN positive cells (red) and GFAP positive cells (green) with nuclear counterstain (blue) in cornu amonis 1 (CA1) and cornu amonis 3 (CA3) of hippocampus. Bars, 25 μm. **(c)** Chao1 diversity indexes of bacterial community and **(d)** relative abundance of bacterial phyla in fecal samples (n=7). Data are presented as mean ± SEM. ^***^*p* < 0.001 indicates significant differences. C: control group; F_1_: 8-week fructose-fed group; AB: antibiotics-treated group.


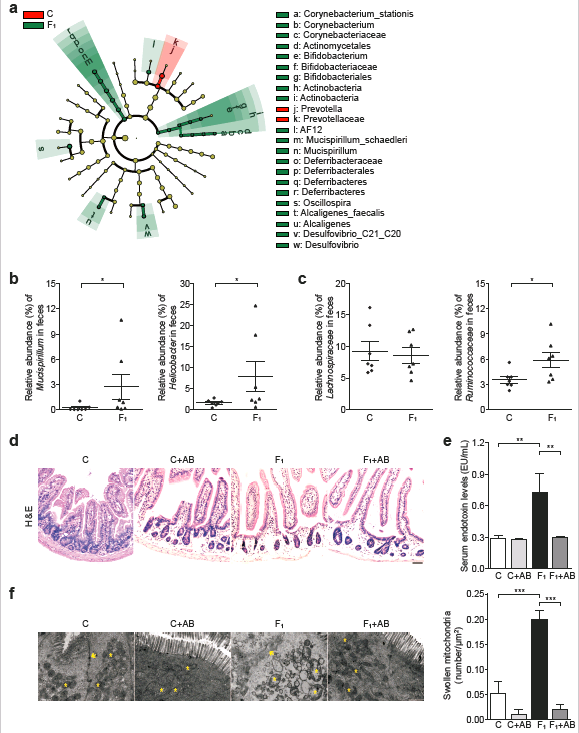


**Additional file 1: Fig S2.** A high-fructose diet causes gut microbial structure alteration and the intestinal epithelial-barrier damage in C57BL/6N mice. **(a)** Cladogram generated from LEfSe analysis (the levels represent, from the outer to inner rings, phylum, class, order, family **(b)** relative abundance of genuses *Mucispirillum* and *Helicobacter* and **(c)** relative abundance of families *Lachnospiraceae* and *Ruminococcaceae* in fecal samples (n=7). **(d)** Histopathology of the distal ileum (Bars, 50 μm). **(e)** Endotoxin levels in serum (n=8). **(f)** Representative transmission electron micrographs of ileum epithelial cells. Arrows indicate gap junctions between two neighbored cells. Asterisks indicate the mitochondria in epithelial cells (Bars, 1 μm). Data are presented as mean ± SEM. ^*^*p* < 0.05 indicates significant differences. C: control group; F_1_: 8-week fructose-fed group; AB: antibiotics-treated group.


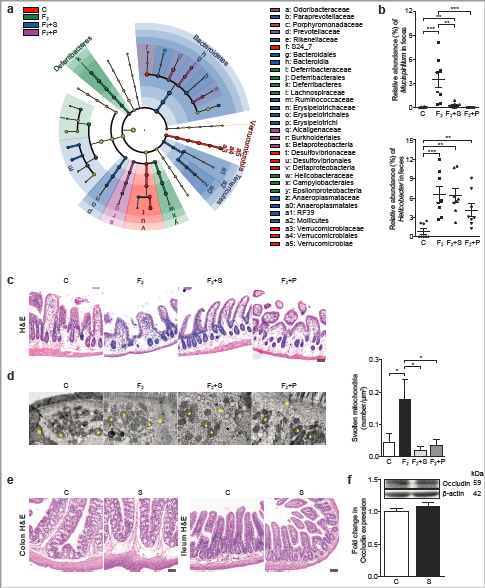


**Additional file 1: Fig S3.** SCFAs and pioglitazone restore gut dysbiosis partly and repair the intestinal epithelial-barrier damage in a high-fructose diet-fed C57BL/6N mice. **(a)** Cladogram generated from LEfSe analysis (the levels represent, from the outer to inner rings, phylum, class, order, family and **(b)** relative abundance of genuses *Mucispirillum* and *Helicobacter* in fecal samples (n=7). **(c)** Histopathology of the distal ileum (Bars, 50 μm). **(d)** Representative transmission electron micrographs of ileum epithelial cells. Arrows indicate gap junctions between two neighbored cells. Asterisks indicate the mitochondria in epithelial cells. Bars, 1 μm. **(e)** Histopathology of the colon and distal ileum (Bars, 50 μm). **(f)** Immunoblot analysis protein levels of Occludin in colon tissue (n=6). Data are presented as mean ± SEM. ^**^*p* < 0.01, ^***^*p* < 0.001 indicate significant differences. C: control group; F_2_: 12-week fructose-fed group; S: SCFAs-treated group; P: pioglitazone-treated group.


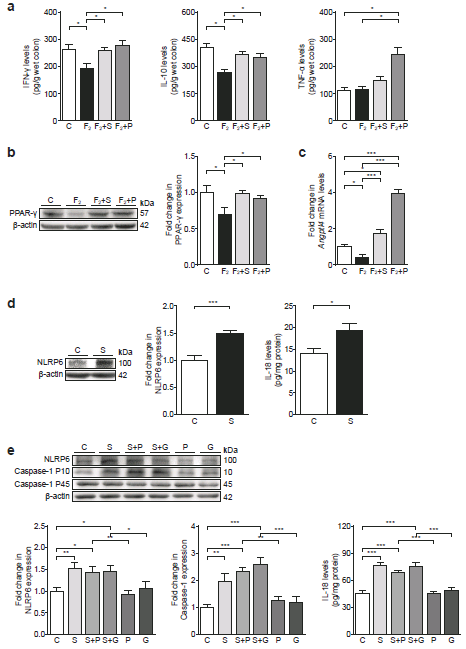


**Additional file 1: Fig S4.** A high-fructose diet impairs gut immune balance and colonic PPAR-γ activation, which is improved by SCFAs and pioglitazone. **(a)** Colonic IFN-γ, IL-10 and TNF-α production normalized to wet weight of colonic tissue (n=6). **(b)** Immunoblot analysis protein levels of PPAR-γ in colon tissue (n=6). **(c)** *Angptl4* mRNA levels in colon tissue (n=6). Immunoblot analysis of NLRP6, Caspase-1 P10/P45 and IL-18 production of **(d)** colonic tissues from the experimental mice (n=6) and **(e)** cultured *ex-vivo* colonic explants (n=6). Data are presented as mean ± SEM. ^**^*p* < 0.01, ^***^*p* < 0.001 indicate significant differences. C: control group; F_1_: 8-week fructose-fed group; F_2_: 12-week fructose-fed group; S: SCFAs-treated group; P: pioglitazone-treated group.


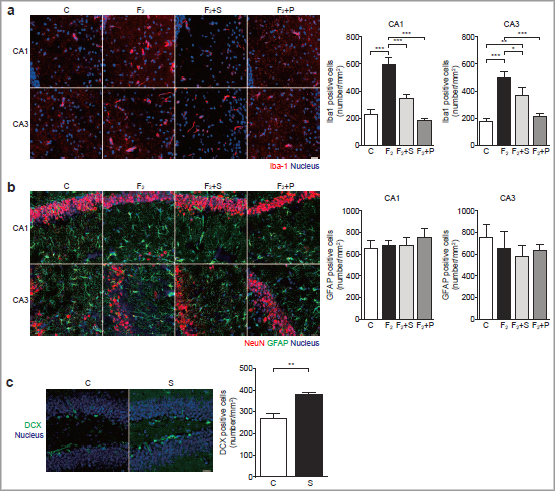


**Additional file 1: Fig S5.** SCFAs and pioglitazone inhibit hippocampal neuroinflammation in a high-fructose diet-fed C57BL/6N mice. Representative immunofluorescence images and quantitative analysis of **(a)** Iba-1 positive cells (red), **(b)** NeuN positive cells (red) and GFAP positive cells (green) with nuclear counterstain (blue) in CA1 and CA3. Bars, 25 μm. **(c)** Representative immunofluorescence images and quantitative analysis of DCX positive cells (green) with nuclear counterstain (blue) in hippocampal DG. Bars, 25 μm. Data are presented as mean ± SEM. ^*^*p* < 0.05, ^**^*p* < 0.01, ^***^*p* < 0.001 indicate significant differences. C: control group; F_2_: 12-week fructose-fed group; S: SCFA-treated group; P: pioglitazone-treated group.


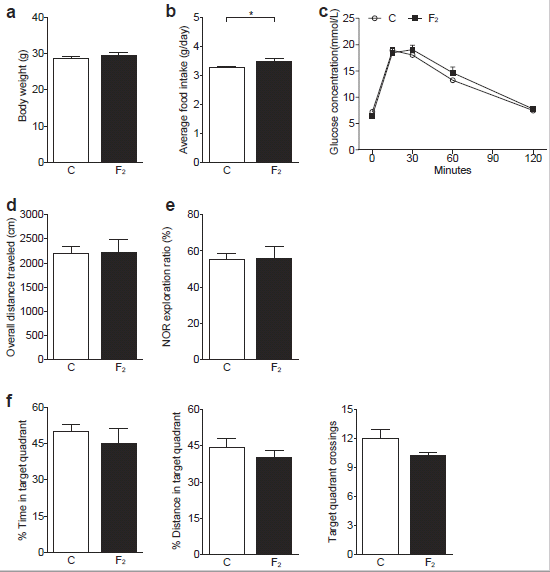


**Additional file 1: Fig S6.** The effects of a high-fructose diet on physiology and behaviors in mice. **(a)** Body weight (n=15). **(b)** Average food intake (n=15). **(c)** The curve of serum glucose in OGTT (n=8). **(d)** Overall distance in the open field test (n=12). **(e)** Exploration ratio in novel object recognition test (n=12). **(f)** The time, the distance and the crossing number of the target quadrant in Morris water maze (n=10). Data are presented as mean ± SEM. ^*^*p* < 0.05 indicates significant differences. C: control group; F_2_: 12-week fructose-fed group.
