# Supplementary material for: Dietary fructose-induced gut dysbiosis promotes mouse hippocampal neuroinflammation: a benefit of short-chain fatty acids
Source: Microbiome. 2019 Jun 29;7:98. doi: 10.1186/s40168-019-0713-7 (PMC6599330; doi:10.1186/s40168-019-0713-7)
Supplement: Supplementary file 2 — Supplemental Methods. (DOCX 24 kb) [file 40168_2019_713_MOESM2_ESM.docx]

**Additional methods**

**Open field test**

Each mouse was placed individually in the middle of an open cubic chamber (25×25×30 cm) for 10 min while its behavior was recorded using a computerized video tracking system and analysis system (Shanghai XinRuan Information Technology Co. Ltd, Shanghai, China). The total distance traveled in the arena was analyzed.

**Novel object recognition test**

This test was conducted as previously described [[1](#_ENREF_1)]. In brief, training, habituation and test sessions took place in a rectangular open-top box (50 × 30 × 35 cm) illuminated by a 25W bulb. Habituation was carried out for 10 min for 2 days during which each mouse was placed individually in the open field in the absence of objects and allowed to explore the environment. Training and test sessions were performed 24 h after habituation. In the first session (5 min) mice explored two identical objects (red glass cylinders 6.6 cm in diameter and 4.5 cm high). During the second session (1 h later), the familiar object was replaced by a novel object (a transparent elongated sphere-like object with an orange cap, 5.5 cm in diameter, 8.5 cm high). Mice were allowed to explore this environment for 5min. Time spent exploring (i.e., sniffing or touching) the familial (T familial) or novel object (T novel) was measured by a trained observer followed by calculation of the exploration ratio [(T familial–T novel)/(T familial + T novel)] ×100.

**Morris water maze test**

Learning and memory ability was performed using the Morris water maze test. The equipment included a round pool (150 cm in diameter and 40 cm in height) and a platform (8 cm in diameter). The pool was filled with water maintained at 20 ± 1 °C and divided into four quadrants with different visual cues on the wall. The platform was placed 1 cm below the water surface in the center of one quadrant.

The test included a 5-day training and a probe trial on day 6. During training, each mouse was trained 4 times with 15-20 min interval a day. A mouse was placed into one of the four quadrants of the pool with its head toward the wall, and drop quadrant was changed each trial. Each trial lasted until the mouse reached the platform and stayed there for 5 s. If the mouse failed to find the platform within 60 s, the trial was ended and the mouse was guided to the platform for 10 s. On day 6, the platform was removed and each mouse was placed into the quadrant that is opposite the target quadrant where the platform was originally placed and allowed to swim freely in the pool for 60 s. A computerized video tracking system (Shanghai XinRuan Information Technology Co. Ltd, Shanghai, China) was used to record the animal's latency to reach the goal. The distance traveled, the time spent and the numbers of crossing in the target quadrant during the trial were calculated by the tracking program.

**Oral glucose tolerance test (OGTT)**

After all the behavioral tests, OGTT was performed. Each mouse was orally administered with glucose (2 g/kg body weight). The tail-vein blood samples were collected at 0, 30, 60, 90 and 120 min after glucose treatment, and then centrifuged (2500 rpm) at 4 °C for 10 min to get serum samples for glucose assay, respectively.

1. Pochwat B, Szewczyk B, Kotarska K, Rafalo-Ulinska A, Siwiec M, Sowa JE, et al. Hyperforin Potentiates Antidepressant-Like Activity of Lanicemine in Mice. Front Mol Neurosci. 2018; 11:456.
